# Unintentionality of affective attention across visual processing stages

**DOI:** 10.3389/fpsyg.2013.00969

**Published:** 2013-12-26

**Authors:** Andero Uusberg, Helen Uibo, Kairi Kreegipuu, Maria Tamm, Aire Raidvee, Jüri Allik

**Affiliations:** ^1^Institute of Psychology, University of TartuTartu, Estonia; ^2^Estonian Academy of SciencesTallinn, Estonia

**Keywords:** EPN, LPP, automatic affective attention, unintentionality, IAPS

## Abstract

Affective attention involves bottom-up perceptual selection that prioritizes motivationally significant stimuli. To clarify the extent to which this process is automatic, we investigated the dependence of affective attention on the intention to process emotional meaning. Affective attention was manipulated by presenting affective images with variable arousal and intentionality by requiring participants to make affective and non-affective evaluations. Polytomous rather than binary decisions were required from the participants in order to elicit relatively deep emotional processing. The temporal dynamics of prioritized processing were assessed using early posterior negativity (EPN, 175–300 ms) as well as P3-like (P3, 300–500 ms) and slow wave (SW, 500–1500 ms) portions of the late positive potential. All analyzed components were differentially sensitive to stimulus categories suggesting that they indeed reflect distinct stages of motivational significance encoding. The intention to perceive emotional meaning had no effect on EPN, an additive effect on P3, and an interactive effect on SW. We concluded that affective attention went from completely unintentional during the EPN to partially unintentional during P3 and SW where top-down signals, respectively, complemented and modulated bottom-up differences in stimulus prioritization. The findings were interpreted in light of two-stage models of visual perception by associating the EPN with large-capacity initial relevance detection and the P3 as well as SW with capacity-limited consolidation and elaboration of affective stimuli.

## INTRODUCTION

The brain processes emotional information in a prioritized manner – a phenomenon known as affective attention. Although this bias exhibits features of automaticity, it is not completely independent of top-down interference. Discovering the specific boundaries of automaticity in affective attention thus remains an important research goal (Vuilleumier and Huang, 2009; Pessoa, 2010). One possibility is that the enhancement of affective content is more automatic during early than later processing stages. Event-related potentials (ERPs) associated with two-stage models of attention (Schupp et al., 2006) are well-suited for investigating this idea. However, existing studies provide not only mixed, but also limited evidence due to their reliance on simplified categorization tasks that limit the depth of emotional processing. In the present study, different ERP components were systematically analyzed to investigate the temporal dynamics of unintentional affective attention during relatively elaborate emotional perception.

To precisely assess the automaticity of affective attention, one first needs to differentiate aspects of automaticity such as effortlessness, unconsciousness, uncontrollability, and unintentionality (Bargh, 1994). For instance, affective attention exhibits a degree of effortlessness since it occurs even during competing task performance (Vuilleumier et al., 2001; Schupp et al., 2003; Keil et al., 2005; Ferrari et al., 2008; Pourtois et al., 2010), as long as some processing resources are left unconsumed by the distracting task (Pessoa et al., 2002; Holmes et al., 2006; Schupp et al., 2007a; Fenker et al., 2009; MacNamara and Hajcak, 2009). Relatively less is known, however, about the independence of affective attention from explicit motivation to process emotional meaning – its’ unintentionality. Existing ERP studies of unintentional emotional processing, analyzed either in isolation (Schupp et al., 2003; Rellecke et al., 2011) or together with intentional processing (Ito et al., 1998; Schupp et al., 2007b; Frühholz et al., 2011; Rellecke et al., 2012; Weinberg et al., 2012), paint an inconclusive picture. More research on the unintentionality of affective attention is thus needed.

Several authors have suggested that automaticity and, by extension, unintentionality of affective attention may vary across processing stages (e.g., Pourtois et al., 2010; Rellecke et al., 2012). A useful framework for conceptualizing this proposal is provided by two-stage accounts or selective (Broadbent and Broadbent, 1987; Chun and Potter, 1995; Marois and Ivanoff, 2005) as well as affective attention (Öhman, 1986; Schupp et al., 2006). According to such models, potentially significant stimuli are first detected at a large-capacity stage, based on crude features of fleeting representations. Selected representations then pass on to capacity-limited consolidation and elaboration stage. In these terms, affective attention involves detection of motivational significance at the first stage that grants prioritized access for emotional stimuli to the second stage (Öhman, 1986; Schupp et al., 2006). Regarding automaticity, capacity differences between the stages suggest that early detection of emotional significance should be more effortless than subsequent elaboration (e.g., Holmes et al., 2006; Pourtois et al., 2010; although see Pessoa, 2010). However, it is empirically (cf. Schupp et al., 2007a; Frühholz et al., 2011; Rellecke et al., 2012) as well as conceptually unclear if a similar temporal difference exists for unintentionality, as this automaticity aspect relates to the presence of top-down goals rather than processing resources. A related question involves the type of interplay between ascending affective and descending intention signals occurring during later processing stages. Do they simply add up (e.g., Ferrari et al., 2008) or somehow interact (e.g., Schupp et al., 2007b; Rellecke et al., 2012) in determining the final prioritization of a given stimulus? The specific aims of this paper are thus to test if early affective attention is unintentional and how intentions subsequently modulate bottom-up bias signals.

Finally, we aim to address these questions in the context of deeper emotional processing than in many previous ERP studies. The effects of intentions are usually studied by manipulating the task-relevance of emotional information. For instance, of all the brain processes observed in a task requiring affective decisions (e.g., “is this a happy or a fearful expression?”), the ones that are also activated by a non-affective task (e.g., “does this face belong to a man or a woman?”) can be considered unintentional. Most previous studies have used binary categorizations tasks like the one just illustrated for this purpose (Ito et al., 1998; Schupp et al., 2007b; Rellecke et al., 2012; Weinberg et al., 2012). However, binary emotional decisions may not induce processing of emotional meaning beyond the first of the two attentional stages. A face can be correctly categorized as happy or fearful based on a binary evaluation (good vs bad) thought to occur at the first processing stage (Bargh, 1994). Reliance on simplified tasks therefore means that existing studies may have failed to consistently induce second-level processing of affective meaning. In the present experiment, a more demanding evaluation task was used in order to elicit deeper emotional processing. Specifically, participants were instructed to evaluate either their personal affective experiences or non-affective stimulus characteristics using polytomous rather than binary response scales.

To address the inter-related aims of this study, we re-analyzed electroencephalography (EEG) responses to emotional pictures viewed in an affective and non-affective task conditions (see Uusberg et al., 2013). ERPs were used to directly assess the intentional and unintentional prioritization of emotional stimuli throughout different processing stages. Affective ERPs reflect the amplification of sensory representations induced by bottom-up as well as top-down mechanisms (Sabatinelli et al., 2007, 2013; Olofsson et al., 2008; Hajcak et al., 2010). Furthermore, we assumed that the early posterior negativity (EPN, 150–300 ms) and late positive potential (LPP, from 300 ms onward) reflect, respectively, the dual processing stages envisaged by two-stage models of attention (Schupp et al., 2006). Based on recent evidence (Weinberg et al., 2012), we also differentiated early P3-like (P3) and later slow wave (SW) sub-components of the LPP. Thus operationalized, the hypothesis that the unintentionality of affective attention is reduced along different processing stages translates into the prediction that affective modulations should be largely independent of task-relevance during EPN, but not during LPP. The pattern of bottom-up and top-down effects on P3 and SW meanwhile should reveal how and when the ascending and descending bias signals become integrated during the second processing stage. Exclusively significant task main effect would indicate a dominant top-down process; a pair of significant main effects would suggest and additive relationship; and a significant interaction would indicate that bottom-up and top-down bias signals have been integrated.

## MATERIALS AND METHODS

### SAMPLE, STIMULI, AND PROCEDURE

The sample analyzed for this paper consists of 79 healthy university students and recent graduates (age *M =* 20.7, *SD =* 2, range 18–29 years; 33 men; six participants discarded due to excessive measurement artifacts). Participants viewed affective images in two conditions – an intentional affect condition where they rated the valence and arousal of emotional states generated by each image; and an unintentional affect condition where they evaluated luminance and object numerocity of the stimuli. Evaluations were collected after each picture on a 9-point scale using a computer keyboard. International affective picture system (IAPS) images from five affective categories – high arousal erotic, low arousal pleasant, neutral, low arousal unpleasant, and high arousal aversive – were selected so that mean normative valence ratings increased from aversive to erotic category, and arousal ratings differentiated erotic and aversive pictures from pleasant and unpleasant ones, as well as the latter from neutral stimuli (see Lang et al., 2005 for normative ratings; and Uusberg et al., 2013 for the image numbers). The stimuli were divided into two sets paired with the two task conditions in a counterbalanced manner. The sets were equivalent in terms of normative affective ratings, semantic content, picture orientation, and luminance. Each set contained 60 pictures (12 from each category). Stimuli from one set were presented in pseudo-randomized order in three blocks with one task instruction before switching to the other set and instruction for another three blocks. The order of tasks was counterbalanced. A single trial started with a fixation cross presented for 1500 ms in the middle of a dark gray screen followed by the stimulus for 1500 ms. Upon stimulus offset two response scales were presented consecutively for unlimited time. All stimuli were displayed on a 14-in computer screen at a distance of 114 cm with an angular size of 15.24° horizontally and 11.52° vertically. A more detailed overview of the data collection methods is available in a study of spectral perturbations within the same dataset (Uusberg et al., 2013).

### EEG RECORDING AND PROCESSING

Continuous EEG was recorded from 30 scalp, four ocular and two earlobe reference electrodes. Offline processing was conducted in EEGLAB (Delorme and Makeig, 2004) and Matlab (MathWorks, MA, USA) software. The data were re-referenced to digitally linked ears, downsampled to 256 Hz, and low-pass filtered at 45 Hz to remove electrical line nose. Infomax independent component analysis (ICA) was trained on 1 Hz high-pass filtered data cleaned of gross artifacts via channel (EEGLAB rejchan; probability; >5 SD) as well as epoch rejection algorithms (rejspec; 20–40 Hz; <-100 and>25 dB). Components capturing eye-blinks as well as horizontal and vertical eye movements were rejected manually for each participant (*M* = 3.6, *SD =* 0*.*87, range 2–6) before reconstructing the continuous, unfiltered data (Debener et al., 2010). The ICA-pruned data were cut into 3000 ms segments covering 1500 ms before and after stimulus onset with -200 to 0 ms removed as baseline. All segments were screened for artifacts using spectral (15–30 Hz, <-30 or >30 dB) and threshold (±100 μV) criteria. If a single channel contained isolated artifacts in more than 2% of trials, the channel was removed before rejecting remaining noisy epochs. On average 90.13% of the data (range 67.5–99.2, *SD =* 7.6%) were retained. The retention rate was independent of affective category [*F*(4,312) = 0.15, *p* = 0.96], task [*F*(1,78) = 0.07, *p* = 0.79], and their interaction [*F*(4,312) = 0.91, *p* = 0.46].

### ANALYSES

We report results from (a) descriptive analyses; (b) removal of physical stimulus confounds; and (c) hypothesis testing. In the first step, affective modulation envelopes (maximum difference between any of the affective and the neutral category at each time point; data from the two conditions collapsed) were plotted at all scalp locations to identify representative time windows for each component of interest – EPN (175–300 ms), early P3-like portion of the LPP (300–500 ms), and later SW (500–1500 ms). Scalp distributions of affective envelopes averaged within these time windows were then used to select representative electrodes for each component (O1, Oz, O2 for EPN and CP1, CP2, P3, P4, Pz for P3 as well as SW). In addition, repeated measures ANOVA of mean valence and arousal ratings of each picture category were conducted to study the stimulus category effects on subjective experiences in the affective condition. In the second step, contributions from physical stimulus characteristics on ERPs were statistically removed to reduce the risk of confounding affective effects with perceptual ones (Carretié et al., 2007; Delplanque et al., 2007). First, the magnitude of this risk was assessed using Pearson correlations between affective ratings and stimulus features on the level of stimulus categories (*n* = 5) as well as single images (*n* = 120; statistical significance testing was omitted due to very different sample sizes). The physical stimulus effects on ERP variability were partialled out using linear regressions for each component. In each regression, single-trial amplitudes were predicted by the luminance and low as well as high spatial frequency (SF) energy estimates of the presented images (obtained from a database of physical characteristics of IAPS images; Delplanque et al., 2007). Residuals from these regressions were considered to be pruned of physical stimulus effects and used for subsequent hypothesis testing. In the final step, repeated measures ANOVAs were conducted for each ERP component with factors for affective category, task, and their interaction. Where applicable, Greenhouse-Geisser corrected *p*-values and Tukey honest significant difference (HSD) *post hoc* test results are reported. In addition, Pearson correlation coefficients were used to directly assess the covariance between subjective ratings and ERP amplitudes across all stimulus categories (*n* = 79 subjects * 5 categories = 395; both types of data collected in the affective condition).

## RESULTS

### DESCRIPTIVE ANALYSES

**FIGURE 1:**
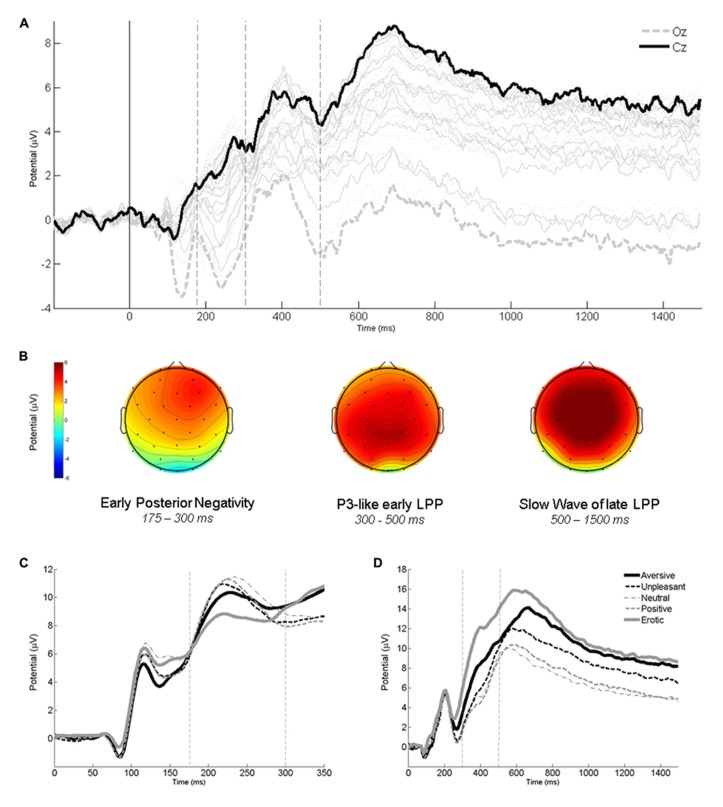
**Descriptive ERP analyses. (A)** Affective modulation envelopes at all scalp locations. **(B)** Interpolated scalp maps of the envelopes averaged within time windows corresponding to EPN, P3, and SW. **(C)** Average waveforms from an occipital region (O1, O2, Oz) showing affective modulation of the EPN. **(D)** Average waveforms from a central-parietal region (CP1, CP2, P3, P4, Pz) showing affective modulations of the P3 and SW. Vertical lines on ERP panels demarcate time windows used for component averaging. All panels depict data averaged across the two intentionality conditions.

The affective envelope clearly indicated temporal boundaries between the three components of interest – EPN and LPP as well as earlier P3-like and later SW portions of the latter (see **Figure 1A**). In the scalp distributions of affective envelopes, the SW extended slightly more frontally than the P3, although both components were strongest over central-parietal electrodes that were selected for further analysis (see **Figure 1B**). Already by visual inspection, expected affective modulations were visible in all ERP components. The EPN exhibited more negativity (see **Figure 1C**) and the LPP more positivity (**Figure 1D**) for most of the affective categories compared to the neutral one.

**FIGURE 2:**
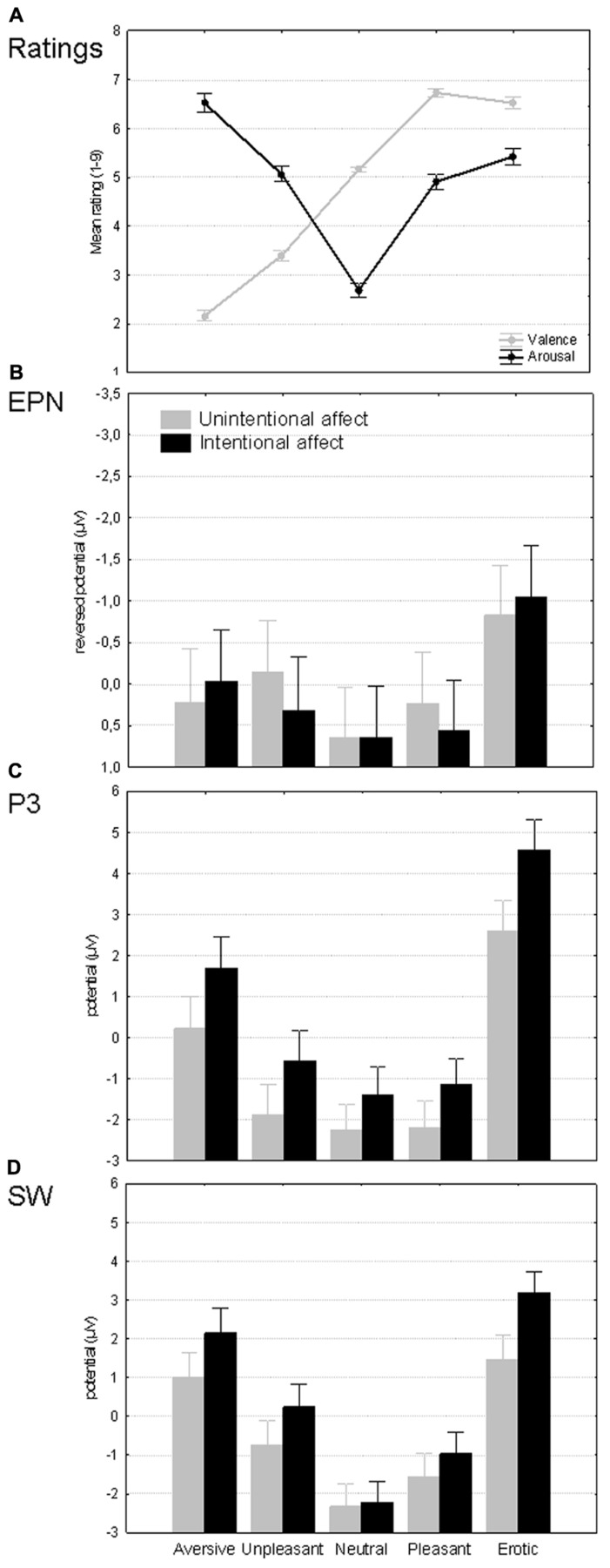
**Overview of the results. (A)** Affective category effects on subjective valence and arousal ratings. **(B–D)** Affective category as well as intention effects on mean amplitudes of EPN (175–300 ms; reversed scale), P3 (300–500 ms), and slow wave (500–1500 ms). Spreads denote standard errors.

Mean affective evaluations of the images are depicted on the **Figure 2A**. Emotional categories differed significantly on both valence [*F*(4,312) = 457.82, _p_η^2^ = 0.85, *p* < 0.001] and arousal dimensions [*F*(4,312) = 133.89, _p_η^2^ = 0.63, *p* < 0.001]. More specifically, valence ratings increased in significant steps (*p* < 0.001) from aversive to pleasant and erotic images, without differentiating the latter two categories (*p* = 0.50). Arousal ratings meanwhile exhibited three distinct levels from lowest neutral via intermediate pleasant and unpleasant (*p* = 0.89) to elevated responses to aversive images (inter-level differences *p* < 0.001). While erotic images were rated to be more arousing than pleasant stimuli (*p* < 0.05), they did not differ from the unpleasant category (*p* = 0.22). Taken together, this pattern confirms intended manipulation of different arousal levels, with erotic images belonging to the second rather than the third level.

### PERCEPTUAL CONFOUNDS

A series of analyses were conducted to assess as well as reduce the possibility that physical differences between stimulus categories would confound substantial inferences. We first confirmed the existence of such a threat by observing that physical stimulus features indeed co-varied with both the dependent (ERP amplitudes) as well as independent variables (affective categories). More specifically, in the regressions conducted to partial out the perceptual variability, physical stimulus features accounted for small but significant amounts of ERP variance (see **Table 1**). Some stimulus features also co-varied with mean affective ratings of stimulus categories (valence and low SF energy *r* = -0.65; valence and luminance *r* = 0.44; arousal and high SF energy *r* = 0.32; other relationships *r* < ∣0.15∣, *n* = 5). Importantly, the co-variance between physical and affective features of the images was confined to the level of stimulus categories – across single images, physical features were independent of the crucial dimension of arousal (*r* < ∣0.10∣) and only minimally related to valence (*r* < ∣0.24∣, *n* = 120). Since the physical confounds were partialled out from single-trial ERP data, this latter observation assures that the removal of perceptual variance did not affect significant amounts of affective variance.

**Table 1:** Contributions of physical stimulus features to single-trial ERP variability.

	EPN		P3		SW	
	F	_p_η^2^	F	_p_η^2^	F	_p_η^2^
Intercept	2326.81	0.083***	823.01	0.031***	1243.28	0.047***
Low SF energy	7.52	0.000**	18.87	0.001***	6.61	0.000*
High SF energy	0.67	0.000	72.46	0.003***	15.41	0.001***
Mean luminance	18.93	0.001***	15.37	0.001***	31.64	0.001***

Whole model R^2^		0.001		0.004		0.002

df = 1,25424; p-values: *<0.05; **<0.01; ***<0.001. SF, spatial frequency; _p_η^2^, partial eta squared.

### HYPOTHESIS TESTING

**Table 2** presents and **Figure 2** illustrates the main results of this paper. The table lists findings from the repeated measures ANOVAs of ERP amplitudes pruned of perceptual confounds. The results pattern was coherent with our hypothesis. The affective modulation of EPN remained completely independent of the task manipulation while P3 and SW amplitudes were determined by both affective category and intentionality in additive and interactive manner, respectively.

**Table 2:** Affective category and intentionality effects on ERP amplitudes.

		EPN		P3		SW	
	Df	F	_p_η^2^	F	_p_η^2^	F	_p_η^2^
Intercept	1,78	0.01	0.00	0.00	0.00	0.00	0.00
Affective category (A)	4,312	16.79	0.18***	110.33	0.59***	63.10	0.45***
Intentionality (I)	1,78	0.14	0.00	27.54	0.26***	8.13	0.09**
A by I interaction	4,312	1.68	0.02	1.58	0.02	2.57	0.03*

Greenhouse-Geisser adjusted p-values: *<0.05; **<0.01; ***<0.001. _p_η^2^, partial eta squared.

The absence of main as well as interactive task effects on EPN amplitudes suggests that the emotional modulations of this component were essentially identical in intentional and unintentional affect conditions (see **Figure 2B**). In both conditions, the erotic category elicited more negativity than any other stimulus type (*p* < 0.001) while both negative categories also exceeded the lowest EPN amplitude level shared by neutral and pleasant stimuli (*p* < 0.01). This pattern cannot be easily explained by either the valence or arousal dimension – no significant correlations were found between EPN amplitudes and self-reported valence (*r* = 0.04, *p* = 0.45) as well as arousal ratings (*r* = -0.06, *p* = 0.25, *n* = 395).

The affective modulation of the P3-like early LPP (see **Figure 2C**) was similar to that of the EPN in the sense that the erotic category again exceeded all others (*p* < 0.001). However, in this component only aversive images rather than all negative stimuli differed from neutral and pleasant pictures (*p* < 0.001). The correlations with ratings revealed that P3 amplitudes had a weak negative relationship with valence (*r* = -0.14, *p* < 0.01) and a positive one with arousal (*r* = 0.11, *p* < 0.05, *n* = 395). P3 was also the first component to be sensitive to intentionality. Specifically, task-relevance of affective meaning increased P3 amplitudes in response to all pictures, including neutral ones. This pattern implies that the P3 was enhanced additively by affective stimulus features and the top-down intention to process them.

Finally, the SW portion of the LPP exhibited yet another affective sensitivity pattern (see **Figure 2D**) whereby most stimulus categories differed significantly from each other (*p* < 0.05) with only aversive and erotic images sharing the highest response level (*p* = 0.18). Like the P3, the SW amplitudes were somewhat related to self-reported arousal (*r* = 0.12, *p* < 0.05) and to lesser extent valence (*r* = -0.09, *p* = 0.07, *n* = 395). The intentionality manipulation meanwhile had an interactive effect on SW amplitudes. The two conditions were matched in terms of responses to neutral images (*p* = 1.00), differed only on trend level for both types of low arousal images (*p* > 0.23) and approached or reached significant differences for high arousal stimuli (*p* = 0.07 for aversive and *p* < 0.001 for erotic). In short, the magnitude of the intentionality effect on SW was roughly proportional with the arousal levels of stimulus categories. This observation suggests that bottom-up and top-down prioritization signals became integrated during the SW window – the extent to which stimuli received additional processing resources when emotions were task-relevant, was modulated by the inherent affective relevance of the images. This dynamics inevitably resulted in slightly different affective modulations within each condition. Most pair-wise differences were significant in both conditions (*p* < 0.05). However, aversive and erotic images elicited similar power levels (*p* = 0.17) in the affective but not in the non-affective condition. Meanwhile neutral images did not differ from pleasant ones and the latter from unpleasant ones (*p* > 0.47) in the non-affective but not in the affective task.

## DISCUSSION

We used ERPs to investigate the unintentionality of affective attention across multiple stages of visual processing. This approach was motivated both by mixed results in existing literature and the lack of research on the unintentionality of deeper emotional processing. We hypothesized that affective stimulus prioritization remains unintentional on the first processing stage and becomes modulated by task-relevance in some particular way on the second. In line with these expectations, the results revealed dissociations between all analyzed ERP components in bottom-up as well as top-down sensitivities. As will be explained in the following sections, these findings suggest that affective attention contributes unintentionally to stimulus prioritization at all analyzed stages of emotional perception, initially without and subsequently with increasingly integrated contributions from top-down signals.

### DIFFERENT AFFECTIVE SENSITIVITIES OF ERP COMPONENTS

Before using them to make inferences about unintentionality, the affective modulations of ERPs observed in this study merit attention in their own right. We observed different patterns in each of the analyzed components: EPN was amplified by all negative and especially erotic images; P3 was enhanced by erotic as well as aversive but not low arousal categories while SW amplitudes differentiated almost all stimuli. These findings resemble earlier reports (Schupp et al., 2006; Olofsson et al., 2008; Hajcak et al., 2010), in particular a recent study of semantically homogenous picture categories documenting strongest ERP responses to erotica and mutilations followed by threating and affiliative content (Weinberg and Hajcak, 2010). This pattern coincides well with the present observation that the erotic and aversive categories dominated most affective modulations. It also helps to explain why the unpleasant category of the present study, containing mostly threating images, amplified ERPs more than the pleasant category, containing only few affiliative depictions. Unlike Weinberg and Hajcak (2010) however, we also found different sensitivities for early and late LPP whereby the latter differentiated high as well as low arousal affective images from neutral while the former responded only to highest arousal, especially to erotica.

These affective modulations can be used to speculate about underlying affective attention mechanisms. Our results suggest that the motivational significance level ascribed to stimuli varies between processing stages, possibly reflecting different amounts of information available at each level. In this framework, the detection system underlying EPN seems to be tuned to potential motivational significance. Having only incomplete representations to work with, subtle distinctions, such as the arousal gradient between aversive and unpleasant stimuli, cannot be identified at this early stage. By the P3 time window, however, sufficient information seems to be available to acknowledge the reduced importance of less arousing unpleasant stimuli. Overall, P3 was sensitive to the unambiguously significant erotic and aversive content without differentiating less arousing emotional stimuli from neutral ones. Finally, four different significance levels were represented in SW amplitudes, suggesting the extraction of emotional meaning was nearing completion. Taken together, these speculations are congruent with two-stage models of affective attention predicting primitive distinctions gradually becoming finer-grained (Öhman, 1986; Schupp et al., 2006). Intriguingly however, the results also suggest that the P3 and SW may reflect sub-stages of the second processing stage, possibly involving preliminary consolidation and subsequent elaboration of stimulus representations.

### UNINTENTIONALITY OF AFFECTIVE ATTENTION

The central aim of this study was to further elucidate the automaticity boundaries of affective attention by specifying when and how top-down intentions interfere with bottom-up detection of motivational significance. The rationale behind the task-relevance manipulation employed to this end was that the intention to evaluate non-affective stimulus features prevented top-down prioritization of emotional features. Thus, the affective modulations that nevertheless emerged in the non-affective condition must originate from bottom-up mechanisms. In this framework, the present findings clearly indicate that bottom-up affective attention contributed to all processing stages, although exclusively only to the earliest one. The intention to perceive emotional meaning meanwhile had an additive effect on the P3 and an interactive one on the SW sub-component of the LPP. Completely unintentional affective modulation of EPN is in line with several earlier reports (Frühholz et al., 2011; Rellecke et al., 2012), although others have documented additive effects on the same component (Schupp et al., 2007b) and even exclusive intention effects for some stimuli (Rellecke et al., 2012). The integration of ascending and descending signals during LPP also converges with many earlier studies (Ito et al., 1998; Schupp et al., 2007b; Frühholz et al., 2011; Rellecke et al., 2012; Weinberg et al., 2012). The shift from additive to interactive nature of this integration between P3 and SW meanwhile is more unique (see also Weinberg et al., 2012).

Some of the discrepancies between the present and earlier results may relate to different decision types used for task-relevance manipulation. We presented participants with polytomous rather than binary response scales in order to induce deeper emotional processing. The differential affective modulations of different ERP components suggest that affective meaning extraction was indeed not completed before the SW window. By contrast, dichotomous decisions required in many previous studies may have only activated the early significance detection underlying the EPN. This difference may explain why the shift from additive to interactive integration of ascending and descending prioritization signals occurred between P3 and SW in this study, but between EPN and LPP in an earlier one using a binary task (Schupp et al., 2007b). Possibly, top-down intention to make a given decision amplifies the bottom-up processing stage where the information required for that particular decision is first extracted. Thus, the binary valence decisions required by Schupp et al. (2007b) may have modulated the EPN because the information necessary for those decisions – basic stimulus evaluations – was encoded already at the first processing stage (Bargh, 1994). By contrast, the information required for the polytomous valence and arousal evaluations of this study might have become available only at the second processing stage. Consequently, the LPP rather than the EPN was amplified by top-down intentions in our results. Future studies should further explore the possibility that top-down task requirements selectively amplify the particular bottom-up processing stage that contains task-relevant information.

More broadly, the intentionality findings of this study clearly support the idea that automaticity of affective attention is reduced along consecutive processing stages. However, the findings also suggest that even while bottom-up bias signals became gradually integrated with top-down ones, the former continued to contribute to stimulus prioritization. More specifically, we found that affective enhancements of stimuli at the first processing stage associated with the EPN were completely unintentional. Early detection of motivational significance can thus operate independent of not only processing resources, but also processing goals. Stimulus enhancements at the second processing stage, meanwhile, were controlled by both ascending and descending prioritization signals. Within this dynamic, a further dissociation emerged between P3 and SW. Specifically, the goal to ignore emotional meaning led to unselective attenuation of all stimuli during the P3, but to selective reduction of only the most arousing representations during the SW. This pattern can be explained by assuming that stimulus processing can be modulated by two partially independent systems – a fast, subcortical affective attention network including the amygdala, and a slower fronto-parietal general-purpose attention network (Pourtois et al., 2013). Using intracranial recordings, the amygdala was recently demonstrated to respond automatically to emotional content between 140 and 290 (cf EPN) before being modulated by top-down attention between 750 and 950 ms (cf SW; Pourtois et al., 2010). In light of these findings, it is possible that the EPN reflects sensory modulations induced mostly by subcortical affective regions while the LPP corresponds to inputs from both the subcortical and the fronto-parietal networks (Sabatinelli et al., 2013). Furthermore, the late-onset top-down modulation of amygdala documented by Pourtois et al. (2010), can explain why affective attention and intentions influenced the P3 additively and the SW interactively in this study. Possibly, the intentions to ignore emotional meaning down regulated only the strong amygdala responses to high arousal stimuli during the SW but not the P3. Even while the anatomical details of this interpretation remain inevitably speculative, our findings are aligned with the broader idea that affective and cognitive attention rely on separate brain networks that can both modulate sensory processing as well as each other.

## CONCLUSION

The findings of this study support two sets of conclusions. First, affective ERP components such as the EPN and the LPP can indeed be interpreted in terms of attention mechanisms operating at two visual processing stages (Schupp et al., 2006). More specifically, our findings can be explained by associating the EPN with fast and largely automatic detection of potential affective significance. The early P3-like and late SW portions of the LPP meanwhile were proposed to reflect, respectively, stimulus consolidation and elaboration within a capacity-limited second processing stage. In addition, the results support the hypothesis that early stages of affective attention are more unintentional than later ones. However, we also observed that affective attention involves unintentional prioritization of emotional stimuli at all processing levels, even while bottom-up bias signals become increasingly integrated with top-down processes. Collectively, the results can be explained by assuming the ERPs to reflect sensory processing that is modulated by competitive inputs from subcortical affective and cortical cognitive attention systems, operating independently at early and becoming integrated at later processing stages.

The present findings also offer some guidance for future studies, which are certainly needed to develop the inevitably simplified associations between specific ERP components with underlying processing stages. First, the direct effects of physical stimulus features on ERP amplitudes are important to control for, whether statistically or by using homogenous stimuli. Our results also suggest that moving beyond simple categorization tasks to manipulate intentions can elicit more substantial processing. We also raised the hypothesis that the stage at which bottom-up processing is influenced by top-down goals may depend on the depth of processing required to satisfy the given intention. Finally, functional dissociations were found between early P3 and late SW portions of the LPP. The present study thus calls for more routine differentiation of these sub-components as well as demonstrates how affective modulation envelopes can help to accomplish this in a data-driven manner.

## Conflict of Interest Statement

The authors declare that there search was conducted in the absence of any commercial or financial relationships that could be construed as a potential conflict of interest.
